# Overlapping multivoxel patterns for two levels of visual expectation

**DOI:** 10.3389/fnhum.2013.00158

**Published:** 2013-04-25

**Authors:** Vincent de Gardelle, Mark Stokes, Vanessa M. Johnen, Valentin Wyart, Christopher Summerfield

**Affiliations:** Department of Experimental Psychology, University of OxfordOxford, UK

**Keywords:** predictive coding, functional imaging, fMRI BOLD, MVPA, repetition suppression, expectations

## Abstract

According to predictive accounts of perception, visual cortical regions encode sensory expectations about the external world, and the violation of those expectations by inputs (surprise). Here, using multi-voxel pattern analysis (MVPA) of functional magnetic resonance imaging (fMRI) data, we asked whether expectations and surprise activate the same pattern of voxels, in face-sensitive regions of the extra-striate visual cortex (the fusiform face area or FFA). Participants viewed pairs of repeating or alternating faces, with high or low probability of repetitions. As in previous studies, we found that repetition suppression (the attenuated BOLD response to repeated stimuli) in the FFA was more pronounced for probable repetitions, consistent with it reflecting reduced surprise to anticipated inputs. Secondly, we observed that repetition suppression and repetition enhancement responses were both consistent across scanner runs, suggesting that both have functional significance, with repetition enhancement possibly indicating the build up of sensory expectation. Critically, we also report that multi-voxels patterns associated with probability and repetition effects were significantly correlated within the left FFA. We argue that repetition enhancement responses and repetition probability effects can be seen as two types of expectation signals, occurring simultaneously, although at different processing levels (lower vs. higher), and different time scales (immediate vs. long term).

## Introduction

Understanding the mechanisms by which visual objects, faces and scenes are recognized is a key question for psychologists and neuroscientists (Logothetis and Sheinberg, 1996). In the visual domain, physiological studies delineating the neural pathways involved in the processing of retinal inputs have described how feedforward and feedback anatomical connections appear to structure the visual brain into parallel hierarchies of processing regions (Maunsell and Newsome, 1987; Felleman and Van Essen, 1991). However, the role played by feedback connections (linking higher to lower regions) in perceptual recognition is still a matter of ongoing investigation. Several theories propose that feedforward signals carrying external information and feedback signals carrying top-down information undergo a process of mutual adjustment to settle on an interpretation of the visual world (Mumford, 1992; Ullman, 1995; Grossberg, 1999; Deco and Rolls, 2005; Friston, 2005). According to one proposal known as “predictive coding” (Friston, 2005), perceptual inference depends on two distinct classes of signal: top-down signals encode predictions about the forthcoming stimulus, while feedforward signals convey the difference between the predicted and observed inputs, a “surprise” signal not dissimilar to the “prediction error” observed for rewards in dopaminergic neurons of the midbrain (Schultz et al., 1997) and the anterior cingulate cortex (Matsumoto et al., 2007). This scheme emphasizes the dual role of expectation and surprise signals in sensory processing, and predicts that these two types of signals should be distinguishable in the visual brain.

Recently, circumstantial evidence for this dual contribution of expectation and surprise signals to the visual brain activity has begun to emerge (Murray et al., 2002; Summerfield et al., 2006; Garrido et al., 2008; Hesselmann et al., 2008; Summerfield and Koechlin, 2008; den Ouden et al., 2009; Kok et al., 2012a). For example, in one recent study using functional magnetic resonance imaging (fMRI), participants passively viewed images of faces and buildings predicted by a probabilistic cue. The blood-oxygen response in an independently-defined face-sensitive region in the fusiform gyrus (FFA; Kanwisher et al., 1997) was best explained by a mixture of “expectation” elicited by predicted faces and “surprise” signals evoked by unexpected faces (Egner et al., 2010), rather than responding to the physical categories alone.

Other studies have used repetition as a tool to manipulate expectation and surprise, under the assumption that during normal viewing conditions, the visual world remains relatively stable, and the presence of a stimulus at one time is a good predictor of its presence in the near future. This intuition provides an explanation for the phenomenon of repetition suppression that is, the reduced neural response to the second or subsequent occurrence of a stimulus (see e.g., Grill-Spector et al., 2006), because repeated (expected) stimuli naturally elicit lower levels of neural surprise than novel, unanticipated stimuli. Under this view, if repetition suppression depends on expectation, then it should be heightened when repetitions are frequent (and expectations are strong) than when they are rare (and expectations are weak). This hypothesis was first tested in a study by Summerfield et al. (2008), who showed that repetition suppression depends on repetition probability, and has since been replicated in a number of studies using faces (Summerfield et al., 2011; Kovacs et al., 2012; Larsson and Smith, 2012), simple shapes (Stefanics et al., 2011), and tones (Todorovic et al., 2011) [although for a failure to replicate with visual objects, see (Kaliukhovich and Vogels, 2011)].

In a recent study (de Gardelle et al., in press) we built upon this framework to provide a stronger test of the view that visual responses are composed of independent signals for expectation and surprise. The reasoning was that over a string of repetitions, expectations should build up, but surprise should be diminished, allowing the potential segregation of voxels that responded negatively (with repetitions suppression indexing the response of putative surprise units) and positively (repetition enhancement indexing the response of putative expectation units) to repetition. Indeed, when participants viewed sequences of 3–4 presentations of a unique face, FFA voxels exhibiting decreasing (repetition suppression, ~65% of voxels) and increasing (repetition enhancement, ~35% of voxels) responses along the sequence constituted two populations that were segregated consistently across runs, responded at differing latencies, and exhibited different patterns of connectivity. These results suggested that repetition suppression and repetition enhancement responses may have distinct functional roles, and perhaps map onto distinct populations of neurons that encode surprise and expectation, respectively.

However, the studies mentioned above—Summerfield et al. (2008) and de Gardelle et al. (in press)—conceive of “expectation” in two theoretically distinct ways. In de Gardelle et al. (in press), the approach followed the classic predictive coding literature with the assumption that expectations arise from *lower-order*, local contextual effects, such as spatial configuration of stimuli (Angelucci et al., 2002; Murray et al., 2002), or in their case, the local temporal structure (i.e., is information repeated or not) (Henson and Rugg, 2003). In other words, repeated stimuli are expected by definition, just as novel stimuli are not. However, in the earlier work by Summerfield et al. (2008), “expectations” were manipulated via the *higher-order* task structure—the probability of occurrence of a repetition or an alternation in prolonged periods of 30–40 trials (repetition probability). In other words, expectations related to the task structure itself, and only indirectly to the likely re-occurrence of any particular exemplar.

The aim of the current study was to used multi-voxel pattern analyses (MVPA) to characterize the relationship between patterns of voxels responding to lower-order expectation elicited by repetition and alternation (either with an enhanced or a suppressed response) and higher-order expectations, elicited by repetition probability. To do this, we collected a new fMRI dataset using a repetition paradigm based on Summerfield et al. (2008) and applied MVPA to assess the consistency across independent observations (i.e., different experimental runs) of the local pattern of voxel-by-voxel responses to repetition effects and repetition probability effects. Participants passively viewed repeating or alternating pairs of faces under conditions where repetition probability was high (80%, i.e., expect repetition) or low (20%, i.e., expect alternation). Rather than using a physical cue to signal repetition probability, which could have confounded MVPA analyses, we simply varied repetition probability over blocks of 10–40 trials that began and ended unexpectedly, under the assumption that participants would learn to expect repetitions or alternations.

This design permitted three distinct approaches to analysing our data. Firstly, we anticipated that the univariate results would replicate the findings of Summerfield et al. (2008) and other studies showing an interaction between repetition effects and repetition probability in the FFA, with stronger repetition effects when repetition probability is high. Secondly, we expected that the multivariate results for repetition would replicate the findings of de Gardelle et al. (in press), showing stable, consistent segregation between repetition suppression and repetition enhancement voxels. Thirdly, we aimed to assess more specifically the similarity between patterns of responses associated with repetition suppression and repetition probability effects, across FFA voxels. Indeed, we reasoned that as much as these two effects are related in time across trials (as indicated by the interaction in the univariate analyses), they might be intrinsically supported by overlapping voxels, and be related in space across voxels. Alternatively, these repetition suppression and repetition probability effects could be mediated by entirely different sets of voxels within the FFA. To distinguish between these possibilities, we measured the cross-correlation between patterns of voxels' responses to repetitions (ALT − REP) and repetition probability (high − low). According to the predictive coding scheme (e.g., Friston, 2005), one would expect this cross-correlation to be positive, which would be interpreted as a greater reduction in surprise responses to novel face stimuli, in voxels that are more sensitive to face expectation signals. If, however, repetition suppression and repetition probability effects were two independent effects across voxels, then we would expect no cross-correlation between their respective response patterns. In other words, the observation of similar response patterns for repetition suppression and repetition probability would suggest that higher order expectation signals (as indexed by repetition probability) and surprise signals (indexed by repetition suppression) are intrinsically related, across voxels.

## Methods

### Participants

Sixteen healthy adults aged between 18 and 37 with no history of psychiatric or neurological disorder, and normal or corrected-to-normal vision, participated in the experiment. Participants gave written consent before the experiment and were paid ~80 Euros for their participation. The experiment was approved by the local ethics committee.

### Stimuli and task

**Figure 1:**
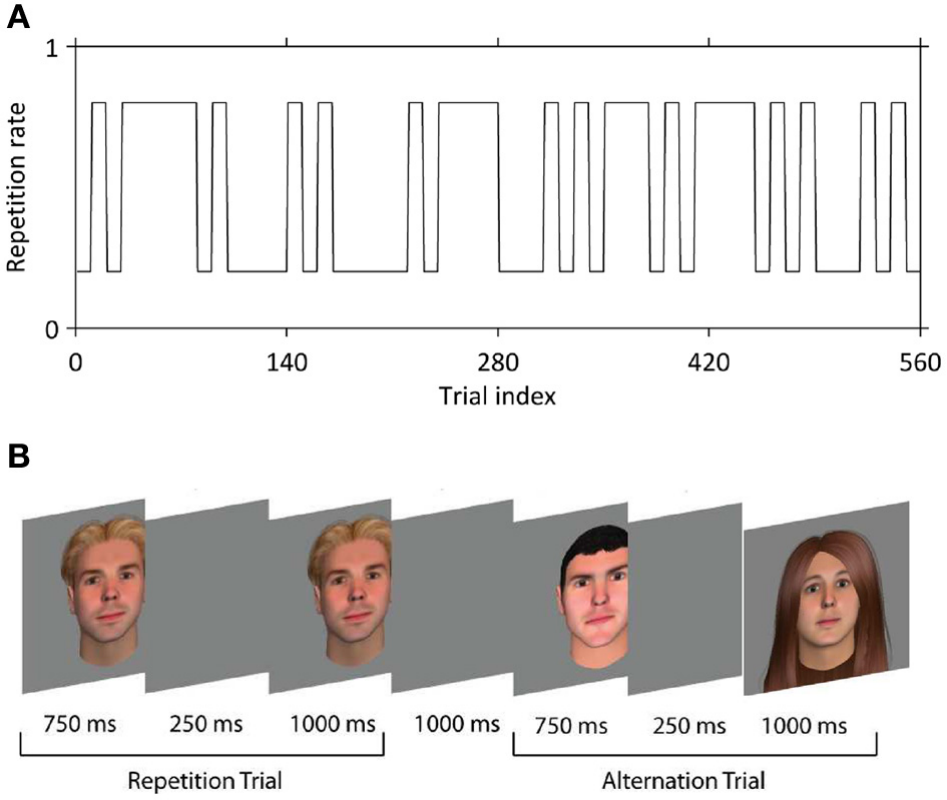
**The behavioral paradigm. (A)** Example of the manipulation of the repetition probability, which over the experiment alternates between 80% (expect rep trials) and 20% (expect alt trials). **(B)** In each trial, two faces were presented in succession that were either identical (repeated trials, REP) or different (alternation trials, ALT).

Face stimuli were 250 × 300 pixel color images of males and females of variable race and age, with hair of differing styles and colors, created using FaceGen (Singular Inversions, Ontario, Canada). Faces were presented centrally on a gray background. On each run of 140 trials (~12 min) participants viewed two faces on each trial, the first for 750 ms, and the second for 1000 ms; the two faces were separated by a blank screen for 250 ms (Figure 1B). Faces were either the same (50% of trials) or different (50% of trials). No face was repeated between trials at any stage of the experiment. In other words, each trial contained either a unique face presented twice (repetitions) or two different unique faces (alternations). Participants completed four runs, i.e., 560 trials in total. A jittered interval with a mean of 3000 ms (range approximately 1000–5000 ms) was interposed between trials. On 17.1% of trials (i.e., 24 per run) either the first face (12 trials per run) or the second face (12 trials per run) rotated slightly to the left or right; this occurred equally often for repetitions and alternations. Participants were instructed to indicate on these trials only (target trials) whether the two faces were the same or different, at onset of the second face. Button presses were made with MRI-compatible response devices held in left and right hands; contingencies were counterbalanced across participants. Trial sequences were constructed such that the probability of a repetition vs. an alternation was set at 20 and 80% every 10 or 30–40 trials (Figure 1A). We verified that participants learned this pattern with a behavioral pilot experiment in which responses were required on every trial. Our experiment was thus a 2 × 2 factorial design crossing stimulus repetition or alternation (REP vs. ALT) with repetition probability (high vs. low). In previous papers, we have referred to this factor as “expectation” but here we use the term repetition probability to avoid confusion with any effect of repetition enhancement which might also be deemed to be due to expectation.

Following the main task, subjects also performed a standard localizer task to define the “fusiform face area” (FFA) (Kanwisher et al., 1997). The localizer consisted of a 1-back task during block-wise presentation of black-and-white photographs (300 × 300 pixels) of faces and houses on a black background, and required subjects to push a button whenever two identical stimuli were presented in a row. Each block consisted of 15 stimuli (including 0–2 repetitions), with each stimulus presented for 750 ms followed by 250 ms fixation, and 10 s fixation periods between blocks. The task consisted of 12 blocks shown in ABAB order (5 min).

### Functional imaging

Magnetic resonance images were acquired with a Siemens (Erlangen, Germany) Allegra 3.0T scanner to acquire gradient echo T2^*^-weighted echo-planar images with BOLD contrast as an index of local increases in synaptic activity. The image parameters used were as follows: matrix size, 64 × 64; voxel size, 3 × 3 mm; echo time, 40 ms; repetition time, 2000 ms. Functional volumes comprised 32 contiguous slices of 3 mm thickness (with a 1 mm interslice gap), ensuring whole brain coverage.

### fMRI univariate analyses

Data were processed using SPM8 (Wellcome Department of Cognitive Neurology, London). The first three volumes of each run were discarded prior to analyses. Preprocessing involved corrections for head motion and slice acquisition timing, normalization to a standard template functional image, re-sampling to 4-mm cubic voxels and spatial smoothing (8-mm full-width half maximum Gaussian kernel). Functional timeseries were high-pass filtered (256 s). The fMRI timeseries were then predicted by regressors of interest coding for discrete events (in the main task) or blocks (in the localizer task) convolved with the canonical hemodynamic response, and by additional nuisance regressors (head motion parameters and their squared values). Temporal correlations were estimated using restricted maximum likelihood estimates of variance components using a first-order autoregressive model. The resulting non-sphericity was used to form maximum likelihood estimates of the activations.

**Figure 2:**
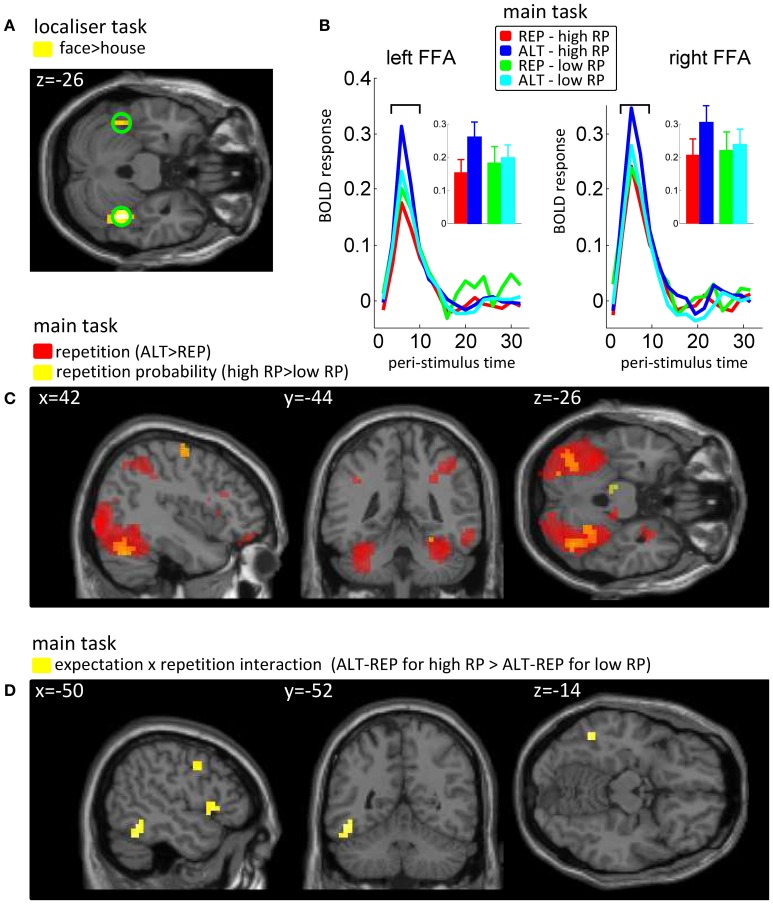
**Univariate fMRI data. (A)** Results from the localizer task, in which the main effect of face vs. house was used to define fusiform face-sensitive regions of interest (FFA). **(B)** Peri-stimuli time histogram of BOLD signals, pooled over voxels in the predefined left and right FFA, as a function of repetition (REP vs. ALT) and repetition probability (high vs. low). Insets show the average BOLD response at bins 3 and 4 (6–8 s post stimulus onset), for the 4 conditions of our design. **(C)** Main effects of expectation (repetition probability, RP) and repetition in the whole brain analyses, reported at a threshold of *p* < 0.005 and *k* = 10. On top of the sagital, coronal and axial views are reported the *x*, *y* and *z* coordinates in the MNI system. **(D)** Interaction between expectation (repetition probability, RP) and repetition. Same conventions as before.

For the localizer task, our regressors of interest coded for onsets and durations of face and house blocks. We then calculated the face > house contrast in each subject and a *t*-test across participants over these images. The peak of this group statistic (Figure 2A) was located at [42, −44, −26] (MNI coordinates). Accordingly, we defined our FFA regions of interest as spheres of 10 mm radius, one on the right hemisphere around this peak, and one at the mirror location in the left hemisphere ([−42, −44, −26]). We note that using individually defined FFA (Table A1) instead of these group FFA regions of interest did not change the results of our analyses (see Appendix).

For the main task, six regressors of interest coded for the two motor responses (left-hand and right-hand button presses), and for the four trial types of our design (REP, high RP; REP, low RP; ALT, high RP; ALT, low RP; where RP denotes repetition probability) when no responses were produced by the participant. Individual contrasts for the main effects of repetition (ALT − REP), and probability of repetition (high − low), and for their interaction, were calculated at the single-subject level and used in simple *t*-tests at the group level. Although our initial hypotheses were specifically associated with the FFA, we also report whole brain results in order to assess whether the profile of results found in the FFA would be also observed in other brain regions. Thus, our whole brain results are exploratory and descriptive in nature. We report the whole brain results at a threshold of *p* < 0.005 (uncorrected) with a cluster extend of 10 voxels, within a global mask formed by brain voxels exceeding half of the maximum value on the grand average functional image. Additionally, we plotted the hemodynamic response (in 16 bins of 2 s) associated with each of the four conditions using a finite impulse response (FIR) filter (Friston, 2005).

### fMRI multivariate analyses

The different multivariate analyses we used were all based on the same pipeline. First, within each block, the raw data timeseries for each voxel were temporally high-pass filtered (256 s) and normalized (zero mean, unit standard deviation). Then, we multiplied the resulting timeseries with the pseudo-inverse of a temporally filtered design matrix. This design matrix was formed by our six regressors (two motor responses, four trial types) convolved with the canonical HRF and by the nuisance parameters. No prewhitening or correction for serial auto-correlation was used. In each run, we computed the contrasts for the main effects of repetition and of repetition probability and their interaction. In each participant we used a leave-one-out cross-validation approach to produce a *Z*-score for each specific test (see details of the different tests below). To do so, we extracted the participant's data in the *N* scanner runs for the FFA, and separated the data into a “selection” dataset that included N-1 runs and a “test” dataset that included the remaining run. Then, the statistic for the test was computed and expressed as a *Z*-score. The leave-one-out procedure was repeated *N* times and the resulting *Z*-scores were averaged, to obtain one value for each participant. Across participants, we then used *t*-tests (one tailed for auto-correlations, two tailed for cross-correlations) to obtain group-level statistics. To preserve the details of the multivoxel patterns, for our multivariate analyses we applied the spatial normalization, re-sampling to 4 × 4 × 4 mm and spatial smoothing routines only after the computing our first-level statistics, and before the group level statistics.

Within this pipeline, we carried out two analyses introduced in our previous work (de Gardelle et al., in press) to assess the separation between voxels showing repetition suppression (i.e., ALT > REP) and voxels showing repetition enhancement (i.e., REP > ALT). Our first analysis assessed the consistency (across scanner runs) of the repetition suppression vs. repetition enhancement segregation in a given region of interest (ROI). Specifically, we counted in each ROI the number of voxels showing repetition suppression in the selection dataset and repetition suppression in the test dataset. This value was converted to a *Z*-score by comparison with a distribution of this value generated under the “null hypothesis.” Here the “null hypothesis” is that the voxels' response signs are sampled independently in the selection and test datasets. Thus, we generated 3000 permutations of the voxels in the test dataset, and counted for each permutation the number of voxels that showed repetition suppression in both the selection and permuted test datasets. This technique ensured that the global responses in the ROI (notably the mean sign in each dataset) were maintained in the “null distribution.” Also, we emphasize that this analysis give the same results if applied to repetition enhancement voxels, because the tendency of repetition suppression voxels to maintain their sign is equal to the tendency of repetition enhancement voxels to maintain their sign, once the mean sign of each dataset is controlled for. From then, we converted the rank of the observed data in the null distribution to a *Z*-score. In the second analysis, we assessed whether both repetition suppression and repetition enhancement voxels would be independently consistent across scanner runs. To test this, in a given ROI we isolated from the selection dataset the voxels showing a repetition suppression profile. Then we calculated for these voxels the auto-correlation of their responses to the repetition contrast between the selection and test datatets. We then applied Fisher's transform to obtain a *Z*-score. This was repeated separately for voxels showing a repetition enhancement profile in the selection dataset. In the whole brain analysis, we looked at the conjunction of the two statistical maps (both thresholded at *p* < 0.005 and *k* = 10).

Our third goal in this study was to assess the similarity between FFA responses elicited by repetition and repetition probability manipulations. To do so, we relied on the cross-correlation between the response patterns corresponding to these two contrasts. Here, we used the average *Z*-score between the two possible situations: using repetition probability responses in the selection dataset and repetition responses in the test dataset, or vice-versa. For completeness, we also calculated the auto-correlation for each contrast (repetition, repetition probability), which would indicate how stable the responses to a given contrast are across separate runs. These analyses are informative because the correlation of two distinct response patterns (here, these associated with repetition and repetition probability) across different runs is limited by the ability of each response pattern to elicit reproducible responses across runs. Finally, we calculated the auto-correlation for the interaction contrast between repetition and repetition probability manipulations, to assess whether the voxels in which the interaction is more pronounced are consistent across scanner runs. Again, all correlation scores were converted to a *Z*-score via Fisher's transform. As an indication, a *Z*-score of 1 here corresponds to a correlation of *r* = 0.08.

### fMRI searchlight

These analyses were initially carried out in the FFA, following previous work and a priori hypotheses that the effects of interest should be present in this region. However, to assess the specificity of our results to this region we also conducted whole brain analyses, using a searchlight or “moving sphere” approach (Kriegeskorte et al., 2006; Haynes et al., 2007; Stokes et al., 2009). There, a 10 mm radius sphere (containing 171 voxels) was defined around each brain voxel, and the *Z*-score is saved at the center of the sphere. This generated a whole-brain statistical map for each multivariate test in each participant. Whole brain results are reported at a threshold of *p* < 0.005 (uncorrected) with a cluster extend of 10 voxels, within a global mask formed by brain voxels exceeding half of the maximum value on the grand average functional image. We have also used a searchlight with a smaller sphere (8 mm radius, about 81 voxels) but the results were essentially unchanged.

## Results

### Behavioral data

Participants responded on 94% of all target trials (misses: mean 5.6 trials, range 0–21 trials). The proportions of missed targets were marginally higher when the trial involved a repetition [4.7% vs. 6.8%, *F*_(1, 15)_ = 3.6, *p* = 0.076], but were not affected by repetition probability or by the interaction (both *p* > 0.4). Similarly, responses on target trials were marginally faster for repetition trials [788 ms vs. 816 ms, *F*_(1, 15)_ = 4.0, *p* = 0.065], but did not indicate any modulation by repetition probability or by the interaction (both *p* > 0.14).

### fMRI univariate results

All fMRI analyses were conducted on no-response trials, to avoid any contamination of our results by motor components. Based on the peak voxel identified in the face > house contrast in the localizer task (Figure 2A), we defined our left and right FFA ROI as two spheres (radius 10 mm) centered at [−42, −44, −26] and [+42, −44, −26] (MNI coordinates). For both ROIs, we extracted peri-stimulus time histograms (PSTHs) of the BOLD response for our four trial types (Figure 2B), and conducted an ANOVA on the amplitudes of the peak of these responses (taking the average of bins 3 and 4, corresponding to 6–8 s after stimulus onset, together to increase signal-to-noise ratio), with repetition and repetition probability manipulations as within participants factors. This analysis confirmed that both the left and right FFA regions of interest were sensitive to repetition [left: *F*_(1, 15)_ = 16.71, *p* = 0.001; right: *F*_(1, 15)_ = 24.78, *p* = 0.0001] and to the interaction between repetition and repetition probability [left: *F*_(1, 15)_ = 6.93, *p* = 0.019; right: *F*_(1, 15)_ = 5.38, *p* = 0.035], with a marginal trend for a main effect of repetition probability in the right FFA [*F*_(1, 15)_ = 3.79, *p* = 0.07].

In the whole brain univariate analysis (at a threshold of *p* < 0.005 and *k* = 10), we found the repetition contrast (ALT − REP) and the repetition probability contrast (high − low) to elicit positive responses in the extrastriate visual cortex, including the fusiform gyrus, although the activations for the repetition probability contrast activations were weaker (Figure 2C). No brain voxels showed a significant negative response to these contrasts. We also computed the interaction contrast (Figure 2D), which notably revealed one cluster in the left fusiform gyrus, with additional clusters in the frontal lobe. These results largely replicate previous findings (Summerfield et al., 2008). Full tables of voxels activated by repetition, repetition probability, and the interaction are shown in Appendix (Table A2).

### fMRI multivariate results: separating repetition suppression and repetition enhancement voxels

Secondly, building upon previous work (de Gardelle et al., in press), we conducted two analyses to assess the separation between voxels showing repetition suppression and voxels showing repetition enhancement. Repetition suppression was found to be highly significant at the group level in univariate analyses, but some voxels might still exhibit repetition enhancement, and our question here, as in de Gardelle et al. (in press), is whether these enhanced responses to repetitions were more likely to be noise (which would not be consistent across scanner runs) or a functionally significant signal (which would be consistent across scanner runs). We began by testing the sign-consistency of FFA voxels, a technique that assessed whether voxels showing a repetition suppression or a repetition enhancement response on a given run were more likely than chance to show a repetition response of the same sign on the remaining runs (see Methods). Indeed, this was the case in the left FFA [*T*_(15)_ = 2.60, *p* = 0.010; one tailed], but not in the right FFA (*T* < 1). Subsequently, we considered the run-to-run auto-correlation for repetition suppression and repetition enhancement populations in isolation, conducting the leave-one-out analysis described above for the repetition contrast separately for repetition suppression and repetition enhancement voxels (see Methods). In our predefined regions of interest, the repetition suppression population was stable in the left FFA [*T*_(15)_ = 4.08, *p* = 0.00049, one tailed] and marginally stable in the right FFA [*T*_(15)_ = 1.53, *p* = 0.074, one tailed], and the repetition enhancement population was stable in the right FFA [*T*_(15)_ = 1.78, *p* = 0.048, one tailed] but not in the left FFA (*p* = 0.19, one tailed).

**Figure 3:**
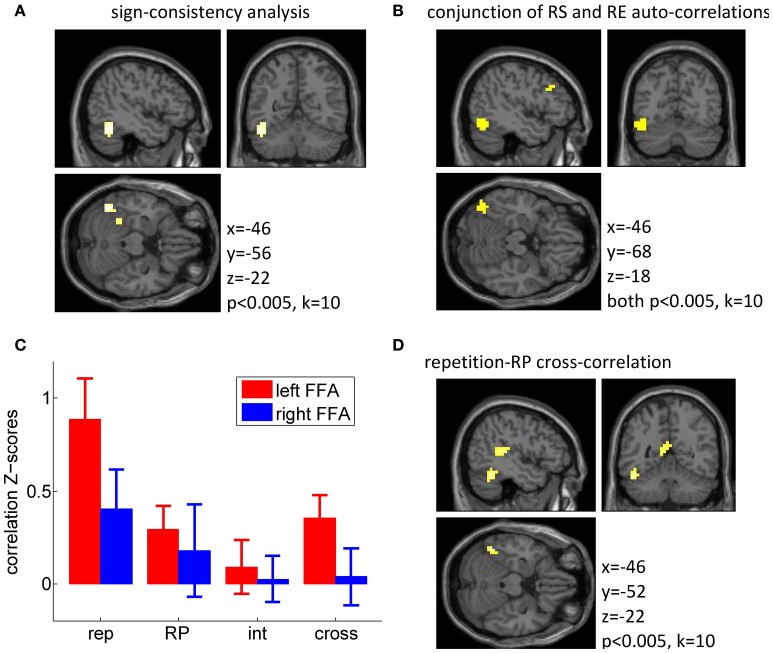
**Multivariate fMRI data. (A)** Searchlight analysis for the sign-consistency measure (see main text for details) indicating significant segregation of repetition suppression (RS) and repetition enhancement (RE) voxels. **(B)** Result from the conjunction analysis showing locations at which separate MVPA analyses for repetition suppression voxels and repetition enhancement voxels both indicated significant repetition-repetition auto-correlation. **(C)** *Z*-scores for across-runs auto-correlations (rep, repetition; RP, repetition probability; int, interaction) and cross-correlation between repetition and repetition probability (cross), within the voxels in the FFA. In this analysis, a *Z*-score of 1 corresponds to a correlation of *r* = 0.08. **(D)** Searchlight analysis results for the MVPA on the cross-correlation between repetition and repetition probability (RP).

We then explored these two effects at the whole brain level using a whole-brain searchlight (Kriegeskorte et al., 2006; Haynes et al., 2007), using a threshold of *p* < 0.005 uncorrected, *k* = 10. This test identified a left fusiform region located slightly more posterior to our FFA region of interest (see Figures 3A,B), as the only cluster responding to the sign-consistency analysis (*k* = 55 voxels; peak: [−46, −56, −22], *p* < 0.001; Figure 3A) and as the main cluster showing simultaneously a significant auto-correlation for repetition suppression and repetition enhancement populations (conjunction: *k* = 34 voxels, center: [−42, −68, −18], Figure 3B). Only one additional cluster was found to exhibit conjunct repetition suppression and repetition enhancement auto-correlations, in the left frontal gyrus (conjunction: *k* = 20 voxels, center: [−38, 16, 22]). These findings, although statistically more modest than those described in de Gardelle et al. (in press), provide a solid replication of those original findings.

### fMRI multivariate results: multivariate patterns for repetition and repetition probability

The MVPA analyses described above indicate that patterns of voxels for repetition suppression and repetition enhancement are independently stable over time. But is a multivariate pattern associated with repetition probability for faces also stable in the FFA, and how does it relate to the pattern associated with the repetition effect? We again used multivariate statistics (correlation *z*-scores; see Methods and Figure 3C) to assess the consistency across scanner runs of the local patterns formed by voxels' responses to the repetition contrast (ALT − REP) and the repetition probability contrast (high − low).

For completeness, we first report the auto-correlations for the repetition contrast and the repetition probability contrast, which confirmed the analyses described above: FFA repetition responses were consistent across runs, with significantly positive auto-correlations both in the left FFA [*T*_(15)_ = 3.98, *p* = 0.00060, one tailed] and in the right FFA [*T*_(15)_ = 1.90, *p* = 0.038, one tailed]. In addition, for the repetition probability contrast, we also found modest but significant auto-correlations in the left FFA [*T*_(15)_ = 2.25, *p* = 0.020, one tailed], but not in the right FFA (*p* = 0.24, one tailed). The responses to the interaction contrast between repetition and repetition probability were not significantly auto-correlated across runs, in either the left or the right FFA (both *p* > 0.27, one tailed), a null result suggesting that the distribution of the interaction effect across FFA voxels may differ across runs.

The more critical analysis was the comparison between the repetition and repetition probability responses in the FFA, which we looked at via the cross-correlation between the two response patterns. If the same population of voxels is sensitive to both manipulations, then there should be a positive cross-correlation between the two patterns. Alternatively, these two effects could be mediated by independent sets of voxels, in which case no cross-correlation should be found. Importantly, we found a significant positive cross-correlation in the left FFA [*T*_(15)_ = 2.92, *p* = 0.010], which indicated that in the left FFA the voxels showing stronger repetition suppression were also more activated by high repetition probability than low repetition probability, i.e., more activated when repetitions were frequent as opposed to when they were rare. The cross-correlation was not significant in the right FFA (*p* = 0.81). The cross-correlation was modest and potentially limited by the noise within each contrast and in particular in the repetition probability contrast, which shows a less reliable auto-correlation than the repetition contrast. Confirming this, we found significant correlations across participants between the individual *z*-scores for the auto-correlations and the cross-correlations, in both FFAs (all *p* < 0.05). In other words, our ability to detect similarity between the repetition and repetition probability contrasts depended on the reliability of both contrasts, and in the present study the repetition probability auto-correlation seems to be the limiting factor.

These findings were corroborated by whole brain results using a searchlight approach (at a threshold of *p* < 0.005 and *k* = 10). The cross-correlation between repetition suppression and repetition probability was significant in a left fusiform region (Figure 3D), but also in several other regions (including the left superior temporal gyrus/BA 41, the precuneus/posterior cingulated/BA 23, and the precentral gyri/BA 4). In addition, the auto-correlation for the repetition probability contrast was found to be significant in one cluster around the right putamen, and in the precentral gyri (BA 4), and the auto-correlation for the repetition contrast was significant bilaterally along the ventral streams, in a very similar manner to the univariate effect, with the additional involvement of bilateral middle frontal gyri (BA 9). Full tables of these results are reported in Appendix (Table A2).

## Discussion

Our study had three main aims. Firstly, we sought to replicate previous studies showing that repetition suppression is modulated by repetition probability in the FFA. We were successful in this regard: univariate analyses showed that repetition suppression (the greater BOLD response to alternation vs. repetition trials) was more pronounced when repetitions were frequent than when they were rare, as would be expected if repetition suppression is at least in part due to attenuation of surprise to a repeated stimulus. This finding replicates our previous work with fMRI (Summerfield et al., 2008), and EEG (Summerfield et al., 2011), as well as other studies that replicated this interaction using auditory (Todorovic et al., 2011), visual (Kovacs et al., 2012), or nociceptive stimuli (Valentini et al., 2011). Our univariate results thus provide further support for theories of perceptual processing that emphasize the combination of higher-order statistics with bottom-up input in visual processing (Mumford, 1992; Ullman, 1995; Grossberg, 1999; Riesenhuber and Poggio, 1999; Llinas, 2002; Rao and Ballard, 2004; Deco and Rolls, 2005; Friston, 2005). However, we note that in the present data the interaction contrast could not be associated with a stable multivariate pattern. Although the reasons for this null effect are not clear, it is possible that it is due to low statistical power in our analysis. On a different note, it is also appropriate to highlight that the reduction in surprise is probably not the *only* mechanism underlying repetition suppression, and it is likely that simpler mechanisms, such as neuronal fatigue, also contribute to repetition suppression. For instance, in the present experiment the main effect of repetition suppression is present with no interaction in early visual regions. Moreover, we note that one study has recently failed to replicate these findings using a different visual stimulus class (objects) (Kaliukhovich and Vogels, 2011). Further work is required using stimuli other than faces to establish the generality of these findings.

Secondly, we expected to replicate the recent finding that voxels exhibiting suppressed and enhanced responses to repetition are stable and consistent across independent observations. In this respect, we were also successful. In the left FFA, both repetition suppression (assumed to index the surprise signals) and repetition enhancement (assumed to index heightened expectation of a given exemplar) elicited stable patterns of responses across voxels, when considered independently. Our sign-consistency analysis tested whether voxels showing repetition suppression vs. repetition enhancement in one run were more likely to exhibit the same response sign in another run. The conjunction analysis assessed whether both the repetition suppression voxels and the repetition enhancement voxels *independently* showed consistent repetition responses across runs. The two analyses provided converging results, and pointed to a left fusiform region that has both properties. This finding replicates our previous results (de Gardelle et al., in press) although with a different design and with arguably a more limited power in the characterization of the repetition effects (in our previous study it was assessed in longer sequences of 1–4 consecutive presentations of the same face stimulus). The significant segregation of repetition suppression and repetition enhancement voxels suggests that both enhancement and suppression of signals by repetition are functionally significant; rather than repetition suppression being the only significant response and repetition enhancement observations being only due to noise. This finding contributes to a growing set of evidence that repetition enhancement responses, which have proved elusive in the past, can be elicited in the visual system (Turk-Browne et al., 2007; Muller et al., 2013). This opens an interesting perspective for future research, which may aim at characterizing the relative functional contributions of repetition suppression and repetition enhancement responses to the processing of novel vs. repeated stimuli.

The third and arguably most interesting objective of our study was to explore the relationship between patterns of FFA voxels responding to repetition and repetition probability manipulations. We reasoned that those voxels most sensitive to lower-order expectation (i.e., alternation vs. repetition trials) might be those which are most strongly modulated by the probabilistic manipulation which determined the higher-order probability of a repetition (high vs. low repetition probability), in which case we should observe a cross-correlation within FFA voxels between the response patterns associated with these two contrasts. This was indeed found to be the case, at least in the left FFA, where we could reject the alternative possibility that repetition and repetition probability responses were supported by independent sets of voxels. In other words, as much as expectation and surprise signals tended to co-occur in time across trials (as indicated by the interaction between repetition suppression and repetition probability in the univariate analyses), they also tended to co-occur in space across voxels, at least within the left FFA. The reason why this result did not extend to the right FFA remains unclear, but a correlation analysis suggests that it could be related to the limited stability of the repetition probability responses in the right FFA.

In the left fusiform gyrus we could thus find three distinct findings: an interaction at the univariate level between repetition and repetition probability, a stable segregation of repetition suppression and enhancement responses (as assessed by both the sign-consistency and conjunct auto-correlation analyses), and a cross-correlation across voxels between repetition and repetition probability effects. Overall, this suggests that in this region both the local expectation signals (repetition enhancement) and the higher order expectation signals (repetition probability and repetition suppression) co-exist. This convergence of results further supports the notion that the generation of surprise responses to novel or unexpected stimuli depends on learning about the statistics of the environment, presumably encoded in a hierarchically superior processing stage. We were not able to identify the source of these learning-based inputs, but one potential candidate region is the right putamen, where the multivariate response was sensitive to the repetition probability. The putamen has long been linked with the formation of new associations during category learning (for a review see Packard and Knowlton, 2002). Moreover, two recent imaging studies identified this region as a critical mediator of the functional connectivity between sensory and motor cortical regions linked by statistical association (den Ouden et al., 2009, 2010). However, whether the putamen mediates the learning about the statistical structure of the task in the present study remains speculative here.

Predictive coding emphasizes that predictions and error signals are passed between multiple levels of the sensory processing hierarchy. Accordingly, our study highlights a distinction between two levels of “expectation,” which are built up over different timescales. At the highest level, expectations are built up about the task sequence—here, critically, the probability of a repetition given the past trial history. One could speculate that these expectation signals, which in our study were observed in the multivariate response in the putamen, might be sent backward to constrain perceptual inference in the FFA. Indeed, in the FFA the reduced responses to repetition were modulated by the repetition probability, and this modulation of repetition suppression could also be observed voxel-by-voxel within the FFA. However, not all FFA voxels are suppressed by repetitions, and repetition enhancement responses potentially indicate a hierarchically lower level of expectation signals, which are generated at the level of the FFA and sent backward to early visual regions (e.g., V1). Thus, one possible interpretation of our data is that two types of expectation signals contribute to the activity observed in the FFA, the first coming from higher regions and constraining the processing of the current stimulus (the repetition probability effect), whose net effect is to boost surprise signals to events incommensurate with the higher statistics of the environment, and the second occurring during or after face recognition, which reflects the accrued information about the stimulus (the repetition enhancement effect) that is then sent backwards to lower visual regions. However, we acknowledge that more research is needed to confirm the feedforward and feedback information flows suggested in this interpretation.

Overall, our results thus contribute to a growing literature that conceives of sensory cortical processing as an economy of expectation and surprise, rather than a mere pre-processing of feature-based information *en route* to higher centers for memory and decision-making. For example, early visual responses are suppressed to stimuli that are predicted (e.g., den Ouden et al., 2009; Alink et al., 2010; Kok et al., 2012a,b). One interesting possibility is that prediction and surprise responses, well-established as characteristic of neuronal activity in the midbrain (Strange et al., 2005) and also observed in limbic structures such as the hippocampus (Matsumoto et al., 2007) and anterior cingulate cortex (Schultz and Dickinson, 2000), are also ubiquitous feature of neocortical processing, even in low-level sensory regions.

### Conflict of interest statement

The authors declare that the research was conducted in the absence of any commercial or financial relationships that could be construed as a potential conflict of interest.

## Appendix Analyses

We confirmed that our analyses revealed identical results when using individually-defined FFA regions of interest, rather than a FFA defined at the group level. The coordinates of the individual FFA are reported on the next page. In summary, all of our results are unchanged with this approach. We report below all the statistical results of these analyses, using the same convention as in our analyses reported in the main text (i.e., the group level statistics were done on Fisher transformed correlation *Z*-scores, and we used one-sided *t*-tests for auto-correlations, two-sided *t*-tests for cross-correlations).

### Univariate analyses

Univariate analyses with individually defined FFA replicated our previous analysis with the FFA defined at the group level. The repetition × repetition probability ANOVA on the peak (bins 3–4) of the BOLD response revealed a main effect of repetition [left: *F*_(1, 15)_ = 30.65, *p* < 0.001; right: *F*_(1, 15)_ = 24.20, *p* < 0.001], as well as an interaction between repetition and repetition probability [left: *F*_(1, 15)_ = 10.23, *p* < 0.01; right: *F*_(1, 15)_ = 8.44, *p* < 0.05]. There was a marginal trend for an effect of repetition probability in the right FFA as well [*F*_(1, 15)_ = 3.53, *p* = 0.08].

### Multivariate analyses: sign consistency and separation of RS and RE voxels

We found significant sign consistency in the left FFA [*T*_(15)_ = 2.93, *p* = 0.005], and a marginal effect in the right FFA [*T*_(15)_ = 1.67, *p* = 0.058]. Then, we assessed the auto-correlation of the repetition effect within RS voxels. Both the left and right FFA exhibited significant effects [left: *T*_(15)_ = 3.75, *p* = 0.001; right: *T*_(15)_ = 2.54, *p* = 0.011]. Within RE voxels, the effect previously found to be significant in the right FFA was now present only as a trend [*T*_(15)_ = 1.32, *p* = 0.103]. As in our previous analysis, there no effect was found in the left FFA [*T*_(15)_ = 0.24, *p* = 0.408].

**Table A1:** **Coordinates for the center of individual FFA (MNI convention, units are mm)**.

	**Left FFA**			**Right FFA**		
	***x***	***y***	***z***	***x***	***y***	***z***
S01	−42	−44	−26	38	−52	−18
S02	−42	−60	−34	42	−60	−34
S03	−46	−48	−26	42	−52	−22
S04	−42	−48	−22	42	−44	−30
S05	−46	−52	−26	38	−48	−26
S06	−38	−36	−22	42	−36	−22
S07	−42	−48	−30	46	−44	−30
S08	−38	−56	−18	38	−60	−22
S09	−34	−48	−38	34	−44	−34
S10	−38	−64	−34	42	−64	−22
S11	−38	−36	−26	38	−36	−26
S12	−34	−48	−38	42	−48	−34
S13	−42	−44	−26	42	−44	−26
S14	−42	−44	−34	38	−56	−30
S15	−42	−48	−30	38	−56	−30
S16	−38	−56	−22	38	−64	−22

**Table A2:** **Whole brain results of the univariate and multivariate analyses**.

	**Cluster**	**Peak**				
	***k***	***Z***	***p (unc.)***	***x***	***y***	***z***
**UNIVARIATE ANALYSES**						
**Repetition effect (ALT − REP)**						
Occipital/fusiform R	974	4.59	0	42	−88	−6
		4.49	0	34	−76	−22
		4.39	0	38	−52	−26
Occipital/fusiform L	781	4.54	0	−38	−84	−14
		4.34	0	−34	−48	−26
		4.25	0	−42	−72	−26
Frontal mid/sup R	52	4.38	0	30	−8	74
		3.52	0	38	−8	58
		3.51	0	30	−4	66
Parahipp L	44	3.98	0	−14	−32	−10
		3.72	0	−22	−28	−14
Fusiform L	14	3.76	0	−30	−8	−38
SMA L	16	3.73	0	−10	8	46
Putamen L	11	3.7	0	−30	12	−2
Parahipp R	35	3.56	0	34	4	−22
		3.49	0	30	−4	−22
		3.24	0.001	18	−8	−18
Paracentral Lobule L	12	3.34	0	−2	−36	74
Frontal Inf R	77	3.28	0.001	54	8	30
		3.19	0.001	58	28	10
		3.11	0.001	46	16	6
Cerebellum R	26	3.25	0.001	14	−28	−22
		3	0.001	18	−32	−34
Medial frontal R/L	11	3.21	0.001	−2	56	−18
		2.59	0.005	6	44	−22
Insula R	12	3.14	0.001	34	24	−2
		2.69	0.004	22	20	6
Middle frontal R	12	3.1	0.001	42	44	−18
		2.78	0.003	38	56	−14
		2.69	0.004	30	40	−18
IPL L	11	3.06	0.001	−38	−44	34
Middle frontal R	13	2.96	0.002	54	32	26
		2.78	0.003	42	28	22
Precentral L	22	2.89	0.002	−42	4	30
		2.82	0.002	−42	−8	42
Parietal supp L	13	2.82	0.002	−26	−72	42
		2.76	0.003	−26	−64	46
		2.71	0.003	−22	−80	46
**Repetition probability effect (High RP − Low RP)**						
Cerebellum/fusiform R	62	4.16	0	30	−52	−22
		3.59	0	42	−68	−26
Pons/brainstem L	18	3.57	0	−10	−28	−30
Occipital sup L	16	3.46	0	−26	−80	22
Cerebellum/fusiform L	34	3.29	0	−38	−72	−30
		3.19	0.001	−30	−64	−26
		3.06	0.001	−26	−76	−18
Precuneus/occipital Sup R	51	3.22	0.001	30	−68	34
		3.21	0.001	30	−68	22
		2.94	0.002	30	−72	46
Precentral R	10	3.1	0.001	42	−12	62
**Repetition × RP Interaction**						
Postcentral R	14	3.59	0	58	−12	26
Precuneus R	17	3.39	0	26	−60	42
SMA L	17	3.27	0.001	−6	20	54
Precentral L	31	3.21	0.001	−34	0	34
		3.21	0.001	−46	0	38
Fusiform L	14	3.09	0.001	−50	−52	−14
Middle frontal L	14	3.08	0.001	−46	28	22
		2.74	0.003	−38	36	22
Inferior frontal R	23	2.99	0.001	50	12	30
Inferior frontal L	16	2.89	0.002	−50	16	2
		2.71	0.003	−38	16	14
**MULTIVARIATE ANALYSES**						
**RP-RP Auto-correlation**						
Putamen/cingulate R	144	4.08	0	30	−8	10
		3.25	0.001	2	−16	42
		2.93	0.002	10	−16	30
Precentral L	98	3.4	0	−30	−16	50
		3.14	0.001	−34	−24	66
Precentral R	73	3.24	0.001	14	−20	82
		2.99	0.001	38	−28	70
Temporal pole L	16	3.11	0.001	−42	16	−34
Precentral L	20	2.84	0.002	−54	0	26
		2.66	0.004	−46	−4	34
**Repetition-repetition auto-correlation**						
Fusiform/occipital L	463	4.29	0	−46	−64	−14
		4.23	0	−46	−52	−14
		3.97	0	−38	−32	−30
Hippocampus L	45	3.61	0	−26	−40	10
Middle/inf frontal L	126	3.6	0	−42	32	38
		3.57	0	−38	20	30
Fusiform/occipital R	300	3.39	0	34	−88	10
		3.24	0.001	42	−72	−26
		3.17	0.001	30	−72	−30
Insula R	44	3.3	0	34	0	22
Middle occipital L	25	2.9	0.002	−26	−96	2
Precuneus L	10	2.83	0.002	−22	−88	46
**Repetition-RP Cross-correlation**						
Insula R	29	3.42	0	34	0	22
Paracentral lobule L	13	3.31	0	−6	−36	54
Cerebellum R	22	3.3	0	−10	−60	−42
STG L	28	3.27	0.001	−42	−40	10
Precentral R	43	3.24	0.001	38	−24	66
Fusiform/cerebellum R	24	3.22	0.001	−46	−52	−22
Precentral R	40	3.08	0.001	14	−16	78
Cerebellum/fusiform L	35	3.07	0.001	−42	−32	−30
Precuneus L	37	3.01	0.001	−6	−56	14
Cerebellum L	10	2.98	0.001	10	−60	−42
Postcentral L	16	2.85	0.002	−34	−32	74
Postcentral R	18	2.76	0.003	14	−40	82

All results are thresholded at p (uncorrected) < 0.005, with a minimum cluster extent of k = 10 voxels. For each cluster, up to 3 peaks are reported, with a localization label (R, right; L, left hemisphere) k, cluster extend; Z, equivalent Z-score at the reported peak; p, uncorrected p-value at the peak (0 indicates p < 0.001); x, y, z are coordinates of the peak (Montreal Neurological Institute system).

### Multivariate analyses: multivariate patterns for repetition and RP

The auto-correlations analyses revealed significant effects for the repetition contrast [left: *T*_(15)_ = 3.88, *p* = 0.001; right *T*_(15)_ = 2.93, *p* = 0.005], for the RP contrast in the left FFA [*T*_(15)_ = 2.54, *p* = 0.011] but not the right [*T*_(15)_ = 0.31, *p* = 0.379] and no significant effect for the interaction [left: *T*_(15)_ = 1.05, *p* = 0.156; right *T*_(15)_ = 0.72, *p* = 0.241]. The cross-correlation between repetition and RP contrasts was significant in the left FFA [*T*_(15)_ = 2.72, *p* = 0.016] but not the right FFA [*T*_(15)_ = 0.79, *p* = 0.441].

### Localization procedure for individual FFAs

For each participant, we calculated the face > house contrast in the localizer data, and used the individual *T*-statistic whole-brain map to define the individual left and right FFA regions of interest. Specifically, we looked for a bilateral cluster around the fusiform gyrus, and for each cluster we took the coordinates of a local maximum close to the fusiform gyrus. For one participant (S13) no obvious cluster was found, so the coordinates of the group-level FFA were used instead.
